# Evaluating the Effect of *Sarconesiopsis magellanica* (Diptera: Calliphoridae) Larvae-Derived Haemolymph and Fat Body Extracts on Chronic Wounds in Diabetic Rabbits

**DOI:** 10.1155/2015/270253

**Published:** 2015-03-18

**Authors:** Jennifher Góngora, Andrea Díaz-Roa, Alejandro Ramírez-Hernández, Jesús A. Cortés-Vecino, María A. Gaona, Manuel A. Patarroyo, Felio Bello

**Affiliations:** ^1^Medical and Forensic Entomology Laboratory, School of Medicine and Health Sciences, Universidad del Rosario, Carrera 24 No. 63C-69, Bogotá, Colombia; ^2^Veterinary Parasitology Laboratory, Faculty of Veterinary Medicine and Animal Husbandry, Universidad Nacional de Colombia, Carrera 45 No. 26-85, Bogotá, Colombia; ^3^Microbiology Laboratory, Faculty of Natural and Mathematical Sciences, Universidad del Rosario, Carrera 24 No. 63C-69, Bogotá, Colombia; ^4^Molecular Biology and Immunology Department, Fundación Instituto de Inmunología de Colombia, Avenida 50 No. 26-20, Bogotá, Colombia; ^5^Basic Sciences Department, School of Medicine and Health Sciences, Universidad del Rosario, Carrera 24 No. 63C-69, Bogotá, Colombia

## Abstract

We evaluated extracts taken from *S. magellanica* third instar larvae fat body and haemolymph using a diabetic rabbit model and compared this to the effect obtained with the same substances taken from *Lucilia sericata* larvae. Alloxan (a toxic glucose analogue) was used to induce experimental diabetes in twelve rabbits. Dorsal wounds were made in each animal and they were infected with *Staphylococcus aureus* and *Pseudomonas aeruginosa*. They were then treated with haemolymph and lyophilized extracts taken from the selected blowflies' larvae fat bodies. Each wound was then evaluated by using rating scales and histological analysis. More favourable scores were recorded on the PUSH and WBS scales for the wounds treated with fat body derived from the larvae of both species compared to that obtained with haemolymph; however, wounds treated with the substances taken from *S. magellanica* had better evolution. Histological analysis revealed that treatment led to tissue proliferation and more effective neovascularisation in less time with both species' fat body extracts compared to treatment with just haemolymph. The results suggest the effectiveness of the substances evaluated and validate them in the animal model being used here as topical agents in treating chronic wounds.

## 1. Introduction

The blowfly* Sarconesiopsis magellanica* (Diptera: Calliphoridae) is of medical interest as it is a potential mechanical vector of pathogens such as viruses, bacteria, protozoa, and helminths [1–3]. Its larvae could produce facultative myiasis without affecting live tissue due to its feeding habits as a necrophagous species, thereby making them useful in larval therapy (LT).

This blowfly is also forensically important as it has been recorded as being one of the first species to colonise decomposing bodies, providing useful information for determining postmortem interval [4, 5].* S. magellanica* has been reported in Ecuador, Peru, Bolivia, Chile, Argentina, and Colombia in South America [6, 7], being found at 1,200 to 3,550 metres above sea level.

*S. magellanica* has been the subject of several recent studies which has led to ascertaining biological characteristics and reproductive and population parameters related to its life cycle in laboratory conditions [8]. Third stage larvae from the family Calliphoridae are characterised as being acephale, having a spiracle-like cephalopharyngeal skeleton; these parts are sclerotised and even though they have a thin cuticle, they are not strongly so [9]. The length of* S*.* magellanica* and* L. sericata* larvae is relatively similar, having an average 11.1 mm [8] and 11.2 mm [10], respectively.

Likewise, this species' proteolytic enzymes have been identified and characterised from excretions and secretions (ES) taken from first, second, and third instar larvae [3]. It has also been shown that this blowfly's larval ES have potent antibacterial activity [11], thereby suggesting the presence of molecules in other larval tissues, having similar functions, which could be of interest in treating chronic and infected ulcers.

The therapeutic use of necrophagous blowfly larvae in treating hard-to-heal chronic wounds (known as larval therapy, biotherapy, maggot debridement biotherapy, or biosurgery) dates back to the beginning of some ancient cultures [12]. However, the first modern approach involving this technology was based on observations made on the battlefield during the First World War by William Baer, an orthopaedic surgeon, who successfully treated patients using larvae from the blowfly* Lucilia sericata* [13]. Nevertheless, this therapy was abandoned with the introduction of antibiotics and surgical advances during the 1940s. Larval therapy underwent a resurgence regarding both clinical and basic and applied research during the last decade of the 20th century as a consequence of bacterial resistance to antibiotics and the need for searching for alternative methods for controlling infection in chronic wounds, thereby promoting important advances which have continued up to today [12].

Identifying and isolating molecules from larval ES as well as those derived from other tissues have been particularly important as they have been evaluated regarding antibacterial control and as promoters of wound healing [14–16]. Different molecular weight antimicrobial peptides (i.e., lucifensin I obtained from* L. sericata* and II defensin from* L. cuprina*) have been isolated and characterised, as have some phenols and lysozymes, acting on pathogenous Gram-positive or Gram-negative bacteria [17, 18]. Such substances are mainly synthesised in fatty bodies, epithelial cells, and certain cells in the haemolymph and then become propagated via this throughout the whole body for contracting infection. The wide distribution of antibiotic-resistant bacterial strains causes infection altering wound healing and such findings are valuable in the search for natural antibiotics for treating chronic wounds [17, 19].

Few studies have dealt with the action of these types of molecule on chronic wounds in* in vivo* models; the role of fat bodies and the haemolymph remains unknown regarding the physiopathology of the processes involved in wound-healing stages using experimentation animal models, highlighting the clinical relevance and reproducibility of the findings. Chronic ulcers are related to underlying diseases, such as diabetes, thereby hindering the favourable evolution of wound healing [20]. Bearing the foregoing in mind, the present work was aimed at evaluating (macroscopically and by using histological techniques) the effect of the* S. magellanica* larvae-derived haemolymph and fat body extracts on experimentally induced chronic and infected cutaneous wounds in diabetic rabbits. The results were compared to other works regarding the same substances taken from* L. sericata* larvae to determine whether they performed the same or produced better effects than* L. sericata* products and validating* S. magellanica* as an eventually useful species in LT.

## 2. Materials and Methods

### 2.1. Obtaining and Sterilising the Larvae

An average of 400 instar II larvae were taken from each blowfly species (*S. magellanica* and* L. sericata*) for each experiment from colonies previously established in the Universidad del Rosario's Medical and Forensic Entomology Laboratory [8, 21]. The larvae were incubated with 4 mL bacterial suspension (OD_620_ = 0.5 nm) consisting of* Staphylococcus aureus* ATCC-29213 and* Pseudomonas aeruginosa* ATCC-27853 at 27°C for 30 min for activating the immune system and potentiating antibacterial activity products [22]. These bacteria are frequently found in chronic wounds [23]. The larvae were then returned to their respective colonies in laboratory conditions to continue their development until reaching instar III stage. The larvae were then disinfected by washing them with 0.5% sodium hypochlorite for 5 minutes with constant shaking, then with 0.5% formaldehyde, and lastly with sterile distilled water.

### 2.2. Extracting the Haemolymph

Two hundred aseptic larvae (used in each experiment) were slightly frozen by exposing them to liquid nitrogen for 10 seconds to immobilise them so that the forward (anterior) part (buccal pieces) could then be removed after having been immersed in phosphate buffered saline (PBS) solution; the haemolymph was collected on a Petri dish which was kept cold (refrigerating gel). It was stored in 2 mL Eppendorf tubes placed on ice and immediately spun at 4,700 g for 40 min at 4°C to separate the cells and enable the plasma (i.e., transparent supernatant) to be collected; this was extracted with a micropipette and filtered through a 0.22 *μ*m membrane (33 mm Millex sterile filter) and stored at −20°C. Before being lyophilised, haemolymph was immersed in liquid nitrogen (10 sec) and then placed in Labconco FreeZone 4.5 equipment at 130 mbars for 15 hours and then stored at room temperature (RT) until being used in treating wounds [17, 24].

### 2.3. Extracting Fat Bodies

Two hundred larvae which had been frozen as described above were taken for each experiment so that the fat bodies could be dissected with the aid of entomological fine forceps and pins. The fat bodies so extracted were stored in 15 mL Falcon tubes cooled on ice; they were then homogenised in a mortar and the extract so obtained was spun at 3,900 g for 50 min at 4°C. This product was stored at −20°C until being lyophilised as described for haemolymph extracts and stored at RT until being used in treating wounds [15, 17].

### 2.4. Animal Model

This study was approved by the Universidad del Rosario's School of Medicine's Ethics Committee, in line with Colombian legislation (Law 84/1989). Twelve healthy male white New Zealand rabbits (*Oryctolagus cuniculus*), weighing an average of 2,500 g and having normal glucose levels (82–90 mg/dL), were used for inducing diabetes. The animals were kept in the Universidad Nacional de Colombia's biotherium in controlled conditions, with 12 : 12 hour light : dark cycle and* ad libitum* access to food and water.

### 2.5. Inducing Diabetes

Alloxan (a thiol reagent) was used to induce insulin-dependent diabetes mellitus; the reagent was dissolved in sterile saline solution until reaching 5% concentration (W/V) and a 125 mg/Kg dose was used on selected animals. The rabbits were anaesthetised with 30 mg/kg ketamine and 3 mg/kg xylazine hydrochloride prior to injecting them slowly with alloxan via the marginal vein in the ear for 3 minutes, using a Jelco Calibre 24 needle. Glucose levels were measured every hour, two hours after the alloxan injection, and up to 12 hours. Animals which were hypoglycaemic (less than 70 mg/dL) were given 20% oral glucose solution to avoid insulin shock, until reaching normal conditions. A glucometer was used for measuring each rabbit's blood glucose concentration 1 to 3 times per day for the experiments. Once hyperglycaemia has been established (two days after the injection), recording greater than 350 mg/dL blood glucose concentration in each rabbit, these values were regulated by administering subcutaneous (SC) insulin according to the following parameters: (1) GL < 400 mg/dL received 1 U/kg, (2) GL = 400–500 mg/dL were inoculated with 2 U/kg, (3) GL = 500–600 mg/dL received 3 U/kg, and (4) GL > 600 mg/dL were given 4 U/kg [25].

### 2.6. Creating and Treating Wounds

Two 2 × 2 cm wide and 0.5 cm deep wounds were made in the skin of each animal (after having been anesthetised), specifically in the dorsum (3 cm between wounds), extending to the adipose panicle. On the following day, 0.5 mL of a solution mixed with bacterial suspension (*Staphylococcus aureus ATCC-29213* and* Pseudomonas aeruginosa ATCC-27853*) at 10^6^ viable microorganisms/mL (0.5 McFarland) concentration was inoculated into each wound. Wound chronicity was determined (day 5) in terms of persistent situations, that is, excessive inflammation and infection, accompanied by clinical signs characteristic for this condition, such as exudate, wound degradation, epithelial bridges, discoloration of the wound bed, abscess formation, and a bad smell. The 12 rabbits were divided into four groups for every assay (all experiments were performed three times independently) after using the same substance according to the following distribution: each wound in group 1 was treated with 0.5 g haemolymph derived from the larvae from both blowfly species (but separately for each wound), group 2 was treated with 0.5 g fat body extract per wound (in the aforementioned conditions), and group 3 was given Furacin (a topical antibiotic), whilst group 4 did not receive any treatment whatsoever and acted as control. Two observers collected the data (simultaneously and independently) during daily follow-up, involving recording macroscopic evaluation regarding wound evolution, following international parameters such as wound bed (WBS) [26] and pressure ulcer scale for healing (PUSH) [27] scores. The evaluations from each session were added and then averaged and interobserver reliability was assessed. The WBS scale evaluated wound healing where the highest total score was the optimum; the parameters evaluated were the presence of sores and/or granulation tissue, wound depth, the amount of exudate, oedema, peripheral dermatitis, calluses, and/or fibrosis around the wounds. Each parameter was scored from 0 (worst score) to 2 (best score) and all scores were then added to obtain an overall score. The PUSH scale was originally used for scoring pressure ulcer evolution [28]. Differently to the WBS scale, the lowest score is the optimum, bearing three aspects in mind: length × breadth, amount of exudate, and type of tissue. Each tool has shown reliability and validity in monitoring and predicting wound healing [26, 28–30]. The average data from WBS and PUSH calculations was carried out by a single researcher in our group.

### 2.7. Histological Analysis

Biopsies were made and processed by histological techniques, following previously described procedure [31]. The first sample was taken on day 5 post-wound induction (PWI); the first 4 mm diameter biopsy was taken from each animal using a dermatological punch. Another two samples were taken on days 12 and 23 PWI. Histological plates were then prepared from each biopsy where the samples were fixed and then haematoxylin-eosin, Masson's trichrome, and Gram stained and viewed under a light microscope. An experienced pathologist having no prior knowledge of each treatment did all later analysis. The results were evaluated taking quantitative variables and wound-healing stages into account (inflammation, proliferation, and maturation). A healthy skin pattern was taken as parameter for the comparative analysis of histological sections. Pain arising from the procedures involved in making wounds was prevented in each animal by the aforementioned analgesia and anaesthesia protocol [16].

### 2.8. Statistical Analysis

The Shapiro-Wilk (S-W) normality test was made, followed by analysis of variance involving a randomised complete block design. Statistica 8.0 software was used for a precise comparison of the treatments. The data was considered statistically significant when the *P* value was <0.05.

## 3. Results

### 3.1. Inducing Diabetes

Diabetes was induced in the rabbits by giving a 125 mg/kg dose of alloxan. There was an increase in the rabbits' blood glucose (sugar) levels (428–531 mg/dL) during the first 5 hours, followed by a fall to a state of hypoglycaemia (57.5 ± 2.3 mg/dL) which persisted for a period of 12 hours. Glucose levels finally increased (511 ± 9.2 mg/dL) and the rabbits became hyperglycaemic until the experiments were completed. The inoculated animals expressed clinical diabetes (hyperglycaemia) and were then divided into two groups according to the value recorded for their glycaemia level: 53.8% (180–250 mg/dL) was classified as being moderate and 46.1% (>250 mg/dL) as severe. No animals died when performing the experiments.

### 3.2. Macroscopic Evaluation of Wounds

#### 3.2.1. WBS Scale

**Figure 1:**
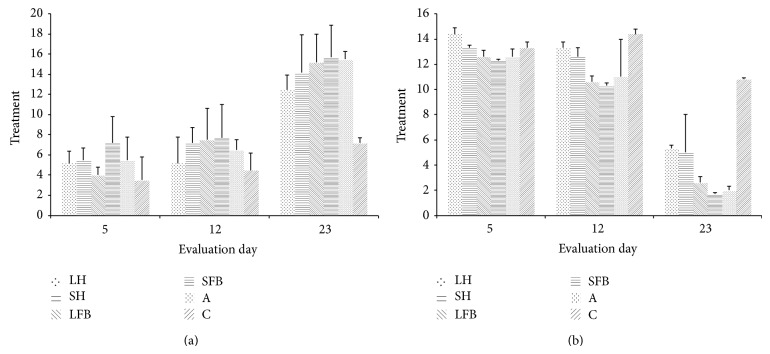
(a) Macroscopic evaluation of healing using WBS scale score on days 5, 12, and 23. (b) Macroscopic evaluation of healing using PUSH scale score on days 5, 12, and 23. LH:* Lucilia sericata* haemolymph-derived larval therapy, SH:* Sarconesiopsis magellanica* haemolymph-derived larval therapy, LFB:* Lucilia sericata* fat body-derived larval therapy, SFB:* Sarconesiopsis magellanica* fat body-derived larval therapy, A: antibiotic, and C: control.

There were statistically significant differences (*P* = 0.0052) S-W (*P* = 0.0000) (*n* = 264) between the groups treated with* L. sericata* haemolymph and the other treatments having higher scores according to the parameters defined for the WBS scale and after evaluating the wounds.  Figure 1(a) shows that as wound healing evolved, the scores for the variables (added together) progressively increased; greater effectiveness was also observed regarding the wounds treated with* S. magellanica* fat body extracts and antibiotic treatment (Figure 1(a)).

#### 3.2.2. PUSH Scale

Evaluating the wounds using the PUSH scale also produced significant differences (*P* = 0.0310) S-W (*P* = 0.0002) (*n* = 264) between wounds treated with* S. magellanica* and* L. sericata* haemolymph and those treated with fat body extracts derived from both blowflies and the group treated with antibiotics; the latter had the lowest scores on this scale, thereby indicating greater effectiveness. Differently to the WBS scale, PUSH scale optimum results regarding treatment with fat bodies reflected lower scores compared to the control; Figure 1(b) shows that as wound healing advanced from day 12 to day 23 PWI, the scores became reduced. Even though there were some differences regarding treatment with each substance used, it was observed that wounds treated with* S. magellanica* and* L. sericata* fat body extracts were more efficient than those treated with haemolymph.

It should be mentioned that biofilm formation could also have been a factor contributing to chronicity; biofilm was detected visually; however, this was not evaluated due to technical limitations.

### 3.3. Treating Chronic Wounds with Haemolymph

**Figure 2:**
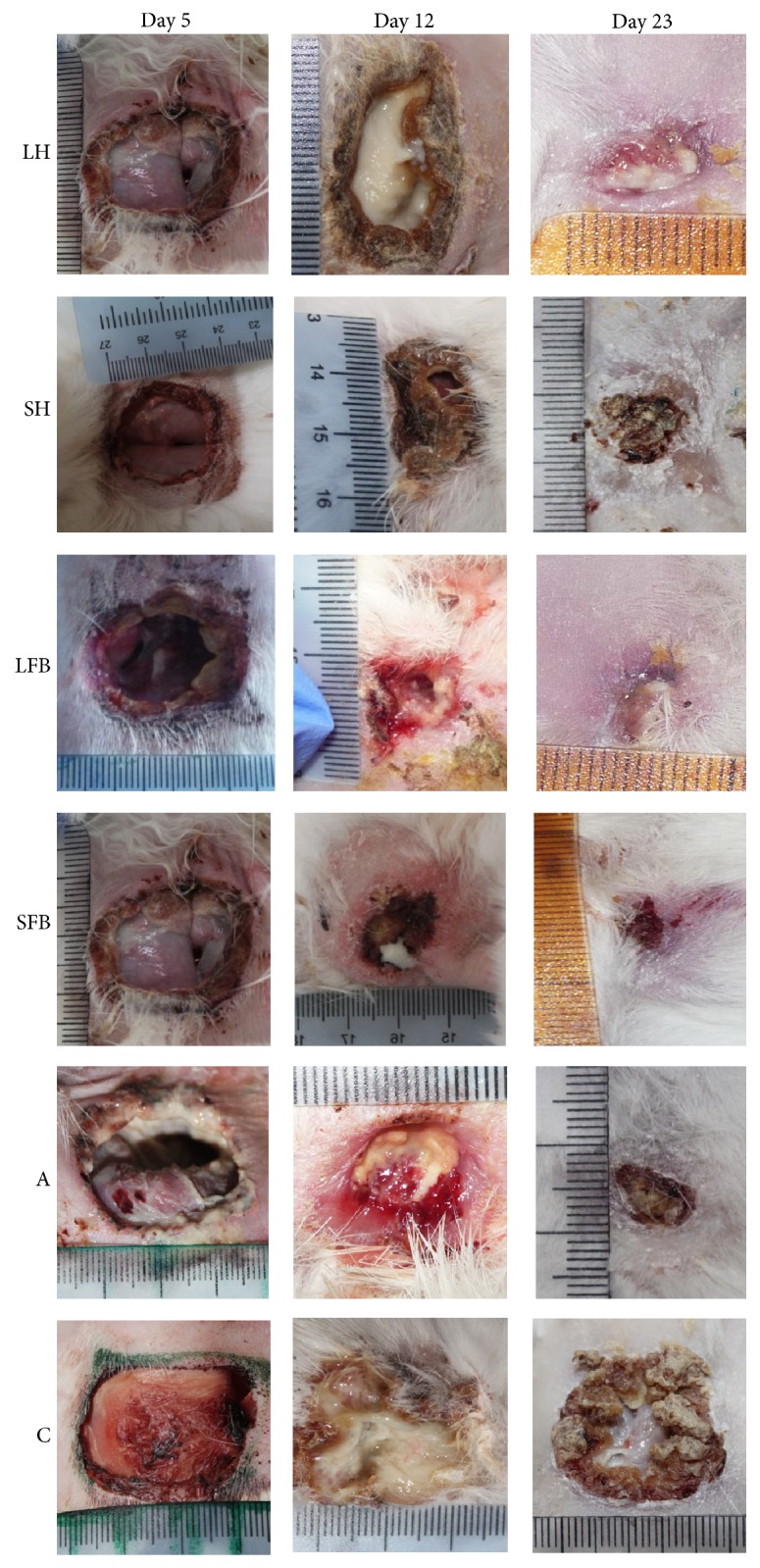
The evolution of diabetic rabbit wounds on days 5, 12, and 23, before and after applying treatment: LH:* Lucilia sericata* haemolymph-derived larval therapy, SH:* Sarconesiopsis magellanica* haemolymph-derived larval therapy, LFB:* Lucilia sericata* fat body-derived larval therapy, SFB:* Sarconesiopsis magellanica* fat body-derived larval therapy, A: antibiotic, and C: control.

The evolution of wound healing treated with haemolymph was established for days 5, 12, and 23 PWI; Figure 2 shows that the evolution of chronic wounds of rabbits treated with* S. magellanica*-derived haemolymph was effective, as wound healing was complete by day 23. This did not happen with wounds submitted to* L. sericata*-derived haemolymph action as the surface of the opening was still evident on the same day PWI (23). Likewise, differences regarding wound healing were more noticeable between days 12 and 23 PWI when comparing control wounds treated with haemolymph (substances extracted from both* L. sericata* and* S. magellanica*).

### 3.4. Treating Chronic Wounds with Fat Body Extracts

Differently to what happened when treating the chronic wounds of rabbits with haemolymph where there was a difference regarding the closing of the rabbits' ulcers, it was noticed that the action of fat bodies was effective regarding wound healing in all the animals evaluated, thereby showing equally optimum results with the substances derived from both blowfly species (Figure 2). This did not happen with the control which maintained the typical characteristics of a chronic wound until day 23 PWI (i.e., remaining in the inflammatory phase).

### 3.5. Histological Evaluation of Chronic Wounds Treated with the Selected Substances

**Figure 3:**
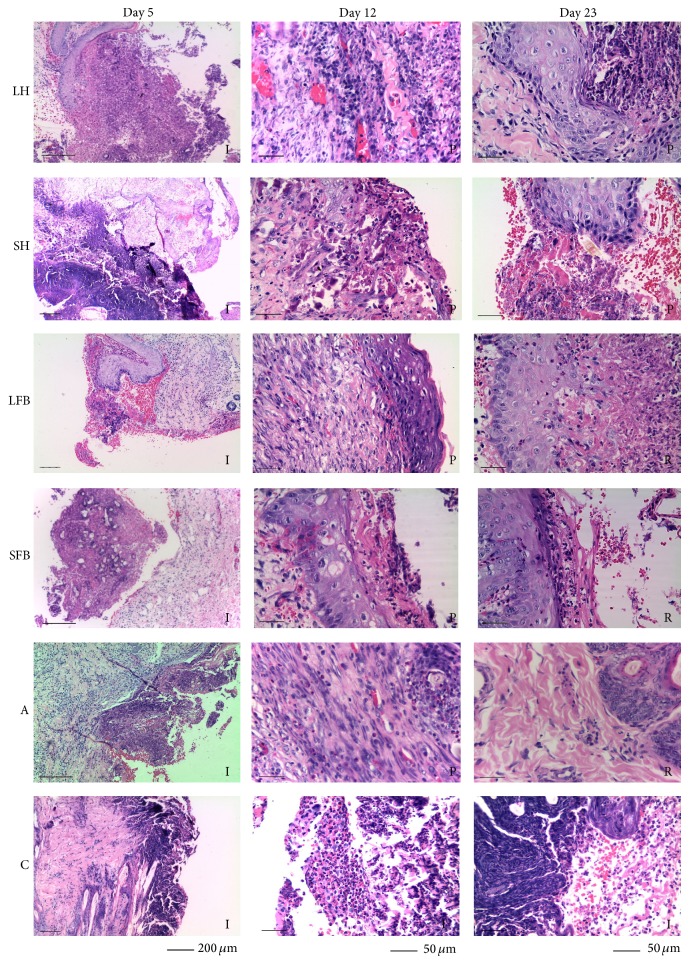
(HE) Histology regarding the evaluation of wounds throughout the healing phases involved in treatment with LH:* Lucilia sericata* haemolymph-derived larval therapy, SH:* Sarconesiopsis magellanica* haemolymph-derived larval therapy, LFB:* Lucilia sericata* fat body-derived larval therapy, SFB:* Sarconesiopsis magellanica* fat body-derived larval therapy, A: antibiotic, and C: control, on days 5, 12, and 23 of the experiments. (I) Inflammation, (P) proliferation, and (M) maturation. The I sections of the figure show abundant inflammatory cells, P the presence of fibroblasts, and M base lamina formation and collagen fibres.

The biopsies and processed tissues were then evaluated by microscope and the samples classified regarding wound/healing stages; the following characteristics were considered: suppurative inflammatory response (with or without bacteria) for the first stage, connective tissue and blood vessel proliferation with the presence of collagen fibres (second stage), and basal lamina and epithelial tissue formation (third stage).  Figure 3 shows the inflammation stage which occurred on day 5; populations consisting of different classes of leukocytes were present.  Figure 3 also shows the presence of connective tissue and blood vessels during the proliferative stage and populations of epithelial cells were typical during the regenerative stage. Wounds treated with fat body extracts evolved in agreement with the characteristics indicated for each wound-healing stage. An inflammatory stage was observed on day 5 when evaluating the wounds (i.e., samples taken before larvae-derived treatment began) where more neutrophils and macrophages were recorded compared to lymphocytes and plasma cells (Table 1). The inflammatory cells were observed before treatment with lyophilised extracts as one of the parameters for verifying wound chronicity. It was established that inflammation in the wounds persisted up to 5 days after they had been induced. Inflammatory cells were also evaluated after treatment on days 12 and 23 where cell counts agreed with more advanced phases of wound healing.

Figure 3 shows wound-healing state according to histological evaluation. This was in the inflammation stage when all treatments began; however, the proliferative stage was recorded as wounds beginning to be closed by day 12 and wounds treated with fat body extracts were established as being in the remodelling stage (third wound/healing stage) by day 23.

## 4. Discussion

The 125 mg/kg dose of alloxan used in the experiments was effective in inducing stable diabetes in the rabbits which exhibited classic symptoms of the disease, mainly hyperglycaemia. There was no need for a second dose, as often happens in some cases when a lower concentration of the reagent is used, for example, 100 mg/kg [25]. Even though alloxan caused selective *β*-cell necrosis in the islets of Langerhans, provoking hypoglycaemia during the first few hours, this was rapidly controlled in the experiments by giving glucose to the rabbits [25] and thus none of the animals died. This method was relatively easy, safe, and practical regarding inducing the base disease in rabbits, even though their susceptibility to diabetogenic dose was not homogeneous and varied, as recorded in previous work [25, 32, 33].

Chronic wounds were successfully induced in the selected animals in a short time, probably due to the combined action of various events, such as the physiopathological effects of diabetes and infection produced by inoculating bacterial strains into wounds, all leading to chronic ulcers. This was important and necessary for evaluating the effect of larvae-derived substances on them in the present work. Inducing chronic wounds in the aforementioned conditions somehow avoided having to wait a longer time for them to appear spontaneously, for example, when human diabetic foot ulcers result from years and even decades of suffering from diabetes mellitus. It was assumed that a combination of various events was needed for the wounds to become chronic in a relatively shorter period of time. Such events would have included the underlying disease, even though being eventually limited regarding its physiopathological manifestations compared to humans, and the persistent infection induced by the bacteria.

According to Wang et al., [25] if a rabbit's lifespan lasts 5 to 8 years, 1 of their years would be comparable to 15 to 20 years of human life. Inducing wounds in hyperglycaemic rabbits and their subsequent infection by selected bacteria accelerated the process even more, until becoming chronic ulcers in a much shorter time. It should be stressed that the animals were maintained with the base disease during the time the experiments lasted (23 days) in a state compatible with life and in reasonably good health. This* in vivo* model for producing infected chronic ulcers is probably the most similar to that suffered by diabetic patients having ulcers on their limbs and although this experimental model cannot mimic all physiological changes affecting wound chronicity in diabetes mellitus, the diabetic rabbit model has been previously validated for evaluating chronic wounds [25, 34, 35].

Larval therapy treatment has been effective regarding this type of ulcer [12, 36]; hence, the model of chronic wounds in diabetic rabbits (used for the first time in this work) proved appropriate and pertinent for evaluating the action of haemolymph and fat body extracts derived from the larvae from the selected necrophagous blowfly species.

The WBS and PUSH scales used here for evaluating wound evolution were seen to be effective tools for following up the healing of chronic ulcers induced in the rabbits. Even though these scales have been used for evaluating chronic ulcers having different origins, the PUSH scale has had greater application and reliability in monitoring wound state and progress [26, 37, 38]. WBS scale parameters regarding treating wounds with fat body extracts (*S. magellanica* and* L. sericata*) and antibiotics had overall scores of 15 to 16, showing satisfactory evolution throughout the days of treatment, accompanied by optimum results.

As* L. sericata* results were relatively better than those for* S. magellanica*, it is worth stating that the WBS scale was considered valid for evaluating and predicting the final closure of wounds [26]. WBS scores thus validated treatment involving fat body extracts whilst the groups treated with haemolymph derived from both blowflies had lower scores (12), indicating that this substance's action led to a proportionally less efficient process. Regarding the attempt to establish macroscopic measurement parameters, the PUSH scale evaluated just 3 factors: wound diameter, the amount of exudate, and tissue type. This led to determining the evolution of wounds receiving different types of treatment. Scores of 2 for wounds treated with* S. magellanica* fat body extracts and antibiotic control were observed up to the final day of evaluation (23), thereby indicating suitable wound healing when the lowest expected values were recorded at the end of the treatment, in spite of high scores on day 12 supporting the inhibition of a diabetic patient's early inflammatory reaction or deteriorated response produced by anomalies regarding granulation tissue formation and collagen production [39]. It is worth stressing that the results obtained here using the WBS scale led to predicting complete wound closure, as reported in previous work [26, 40]. The most notable differences regarding wound healing on days 12 and 23 concerned a reduction in exudate, the appearance of granulation tissue, and a reduction in the size of the wounds. These parameters were determined by PUSH and WBS, agreeing with the days evaluated and wound evolution. The histological results were also taken into account, thereby complementing the calculations made with the aforementioned tools.

Macroscopic observation of wounds treated with haemolymph revealed the characteristics of the inflammatory phase on day 5 of the treatments (Figure 2) by recording necrotic tissue, sphacelus, and abundant exudate, as well as considerable wound size similar to that of negative control (Figure 2). Likewise, there was difficulty in eliminating bacterial infection, probably due to a deficit in endothelial nitric oxide synthase (eNOS) functioning, this being a common anomaly for diabetic patients [41]. Wound healing in normal conditions during inflammatory phase causes an increase of interleukin 1 and alpha tumour necrosis factor which stimulates the synthesis of the aforementioned enzyme and acts as bactericide, especially against* Staphylococcus aureus*. It participates in regulating some cytokines causing a reduction in macrophage migration towards the end of this phase to provide continuity for the proliferative stage [41], contrary to what happens in diabetic patients' wounds and that recorded in our work in diabetic rabbits where there had been previous stagnation during the inflammatory phase. The lesions slowly evolved regarding their macroscopic characteristics on day 12, having remnants from the previous period but the appearance of granulation tissue located towards the edges of the wounds. There were no marked differences regarding the evaluation of wounds treated with haemolymph from both blowfly species, probably due to a similar effect regarding its components' activity. However, wound size became considerably reduced on day 23, mainly for those treated with* S. magellanica* haemolymph, differently to wounds treated with the same substance but derived from* L. sericata*, suggesting that, in spite of the antibacterial properties demonstrated by the haemolymph, the effect was more efficient on those from the first species, similar to the results obtained in a previous* in vitro* study [11]; this evaluated antibacterial activity of larval ES from both species, demonstrating that* S. magellanica*-derived substances were more potent and effective than those taken from* L. sericata*. However, wounds treated with fat body extracts had a greater effect compared to when using haemolymph; for example, a more reduced wound area with abundant granulation tissue around the edges and moderate exudate was observed on day 12 (Figure 2), compared to the effect of treatment involving haemolymph, leading to more favourable evolution by day 23, as there was almost total wound contraction for ulcers treated with fat body from both blowfly species. Such results suggested that unsaturated fatty acids in fat bodies and other organs could act by promoting angiogenesis and wound healing by intervening in several signalling routes [16].

Histological analysis could reveal wound healing; for example, many inflammatory cells were observed on day 5 of wound evaluation, before treatment with larvae-derived substances, which could have indicated vasodilatation produced by activation of the coagulation cascade (haemostasis), accompanied by increased cellular traffic and the release of cytokines (IL-1), tumour necrosis factor (TNF-alpha), and transforming growth factor (TGF-beta) thereby triggering chemotaxis, resulting in the arrival of leucocytes to the wound bed [42]. Neutrophil phagocytic capacity may have become reduced, this being due to a diabetic patient's degree of hyperglycaemia, leading to an increase in these cells' cytoplasmic Ca^++^ concentration [42]. It was seen from day 12 onwards of evaluating wounds with haemolymph from both species that some epithelial cells were possibly migrating towards wounds in an attempt to repair the basal lamina, together with fibroblasts producing type IV collagen, keratinocytes, and plasmid cells; nevertheless, the negative control showed the set of cells from the inflammatory phase. Proliferative phase continued into an advanced stage on day 23 of evaluation with treatments derived from the haemolymph from both blowflies, thus indicating that these substances could induce complete healing later on. Contrarily, Rey et al. [31] demonstrated the total closure of wounds in less than 10 days when evaluating the healing of wounds treated with larval therapy in a nondiabetic rabbit model, even though the therapeutic effect of larvae could have contributed towards the mechanical debridement of tissue due to the larvae crawling over the wounds with the action of their spikes and the digestive effect of the enzymes [12], providing a certain advantage by using live organisms compared to just the chemical action induced by substances extracted from the larvae (haemolymph and fat bodies) as used in the present work. There was also an advantage regarding these animals which could have favoured rapid healing because they did not suffer primary disease (in the aforementioned work), contrary to the diabetic rabbits in the present investigation.

Histological analysis revealed more advanced wound healing involving fat body extract on day 12 of evaluation, differently to wounds treated with haemolymph; there was also evident proliferation of epithelial cells, keratinocytes, and fibroblasts as collagen synthesis and infiltration had begun as well as the appearances of granulation tissue and increased angiogenesis. The present study showed relatively greater advances regarding wounds treated with fat body extracts derived from both blowfly species, probably due to a more effective potentiating effect of unsaturated fatty acids which was related to stimulating synergic activities associated with endothelial cell proliferation and antioxidation [16]. The final stage was established on day 23 of wound healing, accompanied by tissue remodelling and optimum culmination of wound healing concerning wounds treated with both substances derived from both blowfly species and the antibiotic, differently to what happened with negative control which stayed in the inflammatory phase.

## 5. Conclusions

The present work involved inducing chronic wounds in rabbits used for experimental purposes after they had been made diabetic. Both WBS and PUSH scores gave suitable results when evaluating wounds, reflecting advances in wound evolution; the PUSH scale proved relatively simpler and more practical to use. A better score was obtained for wounds which had been treated with fat body extract, probably due to unsaturated fatty acids' metabolic action, even though there was better performance regarding treatment with* S. magellanica* extracts. Histological analysis at three different times revealed more notable characteristics concerning wound healing stages, as the results showed more advanced wound healing with treatment involving fat bodies compared to haemolymph. The foregoing led to concluding that the substances evaluated concerning chronic wounds in the animal model used here were effective regarding wound-healing treatment, even though they have some differences, and larval fat body-derived substances were relatively more effective.

## Figures and Tables

**Table 1:** Average cell count in diabetic rabbits' chronic wounds. Inflammatory phase, before starting treatment, quantified in 15 random fields.

	LH	SH	LFB	SFB	A	C
Neutrophils	149 ± 51.2	149.5 ± 72.8	221.2 ± 7.8	189.7 ± 3.0	169.2 ± 4.7	176.6 ± 6.7
Macrophages	70.5 ± 18.7	113.5 ± 9.4	148.5 ± 62.1	29.5 ± 12.1	122 ± 6.1	144.6 ± 13.0
Lymphocytes	12.75 ± 2.7	49 ± 1.8	43.2 ± 8.4	25.5 ± 9.5	32.5 ± 3.6	20 ± 3
Plasma cells	32 ± 5.3	35.25 ± 12.9	44 ± 17.8	14 ± 5.2	23 ± 3.9	11.6 ± 4.7

LH: *Lucilia sericata* haemolymph-derived larval therapy, SH: *Sarconesiopsis magellanica* haemolymph-derived larval therapy, LFB: *Lucilia sericata* fat body-derived larval therapy, SFB: *Sarconesiopsis magellanica* fat body-derived larval therapy, A: antibiotic, and C: control.
